# Immediate changes in stroke patients’ gait following the application of lower extremity elastic strap binding technique

**DOI:** 10.3389/fphys.2024.1441471

**Published:** 2024-09-11

**Authors:** Yuduo Liu, Qi Wang, Qiujie Li, Xueji Cui, Huimeng Chen, Xianglin Wan

**Affiliations:** ^1^ Biomechanics Laboratory, Beijing Sport University, Beijing, China; ^2^ Key Laboratory for Performance Training and Recovery of General Administration of Sport, Beijing Sport University, Beijing, China; ^3^ People’s Hospital of Queshan, Zhumadian, China

**Keywords:** stroke, gait, lower extremity elastic strap binding technique, statistical parametric mapping, joint angles

## Abstract

**Objective:**

To ascertain the immediate changes in stroke patients’ temporal and spatial parameters of gait and the joint angles of stroke patients throughout the entire gait cycle following the application of lower extremity elastic strap binding technique.

**Methods:**

Twenty-nine stroke patients were invited as the study participants. The patient seated, flexed the hip and knee, utilized a 5 cm-wide elastic strap, positioning its midpoint beneath the affected foot and crossing it anterior to the ankle joint. Upon standing, the strap encircled the posterior aspect of the lower leg, proceeded around the back of the knee, and ascended the thigh on the affected side. Crossing anteriorly over the thigh, it then encircled the back of the waist before being secured in place. Using Qualisys motion capture system to collect kinematic data of the lower extremities during walking while wearing shoes only or strapping. A paired sample t-test was used to analyze the effects of the technique on gait spatiotemporal parameters and joint angles in stroke patients.

**Results:**

The patients’ step length decreased (*P* = 0.024), and step width increased (*P* = 0.008) during the gait cycle after the strapping. In the gait cycle between 0% and 2%, 7%–77%, and 95%–100%, the hip flexion angle on the affected side was significantly larger after the strapping (*P* < 0.05). In the gait cycle between 0% to 69% and 94%–100%, the knee flexion angle on the affected side was significantly larger after the strapping (*P* < 0.05). In the gait cycle between 0% to 57% and 67%–100%, the ankle dorsiflexion angle on the affected side was significantly smaller after the strapping (*P* < 0.05), and in the gait cycle between 0% to 35% and 68%–100%, the ankle inversion angle on the affected side was significantly smaller after the strapping (*P* < 0.05).

**Conclusion:**

The lower extremity elastic strap binding technique can decrease the hip flexion and knee flexion limitations in stroke patients during walking, and reduce the ankle plantar flexion and ankle inversion angle of stroke patients. The lower extremity elastic strap binding technique enabled stroke patients to adopt a more stable gait pattern.

## 1 Introduction

Stroke is one of the leading causes of adult death and long-term disability in China (Katan and Luft, 2018; Zhou et al., 2019). More than 50% of patients have movement disorders such as walking difficulties after a stroke, which seriously affects the quality of patients’ lives (Mahlknecht et al., 2013). Reduced physical activity due to the inability to walk properly increases the risk of developing secondary health conditions (Rodríguez-Fernández et al., 2021), such as respiratory and cardiovascular complications, bowel/bladder dysfunction, obesity, osteoporosis and pressure sores, which further reduce the life expectancy of patients (Booth et al., 2012). Therefore, the restoration of independent walking ability is one of the main rehabilitation goals for stroke patients (Rodríguez-Fernández et al., 2021).

There are many traditional gait rehabilitation methods for stroke, and some new methods are also being proposed, some of which have unclear effects. Wearing ankle-foot orthoses has been proved to affect gait biomechanics (Brown et al., 2017). However, due to low comfort and high economic cost, orthoses have not been widely used in clinical practice in China (Dong et al., 2020). In recent years, scholars have designed strap binding technology which is more comfortable and lower-cost (Hwang et al., 2013). It uses elastic bandages to wrap around different parts of the patients’ limb in an attempt to use the elastic characteristic of the strap to stabilize and support the various joints and the weaker muscle groups, thereby improving the abnormal gait of stroke patients. There are many ways to bind the strap, such as a single bilateral method for mild foot drop, insufficient bilateral hip extension, and poor trunk stability (Liu et al., 2022; Liu et al., 2023). A single, unilateral method for correcting ankle inversion and external rotation of the hip (Liu, 2017; Wang et al., 2018; Liu et al., 2022; Liu et al., 2023), and a combination of different methods (Wang et al., 2018), etc. As one of the single unilateral methods, the lower limb anterior rafting strap binding technique binds the joints of the lower limbs and finally fixes the strap on the shoulder, providing forward and upward assistance to the lower limbs of the hemiplegic side, while increasing the abdominal pressure of the patient and enhancing the core stability (Wang et al., 2018). In order to improve the ability to walk independently, stroke patients should relearn movement patterns by practicing walking repeatedly (Mayr et al., 2007). Thus, if rehabilitation therapy yields immediate improvements in gait for stroke patients, it could be considered as a viable long-term intervention. The lower limb anterior rafting strap binding technique is widely used in clinical practice. However, in the existing studies, there is no report on the immediate effect evaluation of the lower limb anterior rafting strap binding technique that only binds the affected lower extremity.

It has been found that strap binding techniques could improve patients’ scores on the Fugl-Meyer scale, Berg scale and Barthel index (Zhang et al., 2016). However, the above scale-like assessment methods for evaluating lower limb function, balance function, and daily activity ability often need to be implemented by experienced doctors or occupational therapists, thus the results are subjective to a certain extent. Some more objective methods, such as analyzing the human kinematics parameters when the patient completes the movement, will help to further evaluate the rehabilitation effect of this technology (Xu et al., 2022), but there is relatively little research in this area. The study found that the strap binding technique increased the step length (Hwang et al., 2013), increased the step width (Zhang et al., 2016), increased the peak moment of hip and knee flexion (Wu et al., 2017), and reduced the ankle inversion angle (Xie et al., 2013) during the patient’s walking. However, these studies all use discrete point analysis, usually focusing on peak indicators, which may introduce bias in data extraction. Statistical Parametric Mapping (SPM) is a statistical method used to analyze the difference between the entire curve rather than a single peak (Pataky et al., 2013), which can further quantify the influence of the lower limb anterior rafting strap binding technique on gait characteristics throughout the gait cycle.

Therefore, the objectives of this study are as follows: 1. To ascertain the immediate changes in stroke patients’ temporal and spatial parameters of gait following the application of lower extremity elastic strap binding technique. 2. To evaluate the immediate changes of the lower extremity elastic strap binding technique on the joint angles of stroke patients throughout the entire gait cycle. Based on the above two objectives, we aim to provide a theoretical basis for the selection of gait rehabilitation measures for stroke patients. Based on the way of the strap binding, this study hypothesized that the lower limb anterior rafting strap binding technique may result in the following changes: 1. Decrease in step length, increase in step width, and an increase in the proportion of stance phase on the affected side, a decrease in the proportion of stance phase on the unaffected side, and a reduction in the unaffected-to-affected side stance phase ratio for stroke patients. 2. Increase in hip flexion angles, decrease in hip abduction and external rotation angles, increase in knee flexion angles, and a decrease in ankle dorsiflexion angles during walking compared with wearing shoes only.

## 2 Material and methods

### 2.1 Experimental participants

Inclusion criteria: ①The patients were diagnosed confirmed clinically by computed tomography or magnetic resonance imaging (Hyun et al., 2015); ②Functional Ambulation Category Scale (FAC) rated level 3 or above; ③Vital signs are stable and there are no other diseases that may affect gait; ④Clear awareness and the ability to understand basic commands; ⑤Signed informed consent; ⑥Age range: 40–70 years old.

Exclusion criteria: ①Patients with a previous clinical diagnosis of Parkinson’s disease, fracture, myasthenia, or other diseases affecting gait; ②Patients with severe cognitive impairment who are unable to follow instructions.

G*Power 3.1.9.7 software for Windows (Heinrich-Heine-Universität Düsseldorf, Düsseldorf, Germany) was employed to conduct an a priori power analysis to determine the appropriate sample size with 80% power, effect size of 0.4 and an α error probability of 0.05. A nondirectional (two-tailed) analysis was applied. According to the aforementioned parameters, the sample size of 26 participants is needed to achieve the desired statistical power. A total of 29 stroke patients (25 males; 4 females) were invited as participants, and the specific information is shown in Table 1. This study was approved by the Ethics Committee of Beijing Sport University (approval document No. 2021080H).

**TABLE 1:** Baseline data of the participants.

	sample size(n)	age(y)	height (cm)	body mass (kg)	left affected limb(n)	right affected limb(n)	FAC level
males only	25	52.8 ± 9.3	168.7 ± 7.4	73.9 ± 7.1	12	13	4.2 ± 1.0
females only	4	55.3 ± 5.0	158.3 ± 6.1	66.7 ± 11.7	3	1	4.0 ± 0.8
all participants	29	53.1 ± 8.8	167.2 ± 8.0	72.9 ± 8.1	15	14	4.1 ± 0.9

Note: FAC, functional ambulation category scale.

### 2.2 Methods

#### 2.2.1 Protocol

The participants wore uniform tights and brought their own sports shoes. A total of 19 passive reflective markers were placed bilaterally on each participant’s lower extremity, including the anterior superior iliac spine, the anterior thigh, lateral and medial femur condyles, lateral and medial malleolus, tibial tuberosity, the heel, middle of the 2nd and the 3rd metatarsals. An additional marker was attached at the junction of the 4th and 5th lumbar spine vertebra (L4 and L5). Before and after the strapping, the participants walked on the ground for about 10 m at their comfortable speeds respectively. The participants walked to the end point, and the coordinates of all body surface markers were collected as a valid collection. The valid data were collected three times in the two states, respectively.

**FIGURE 1:**
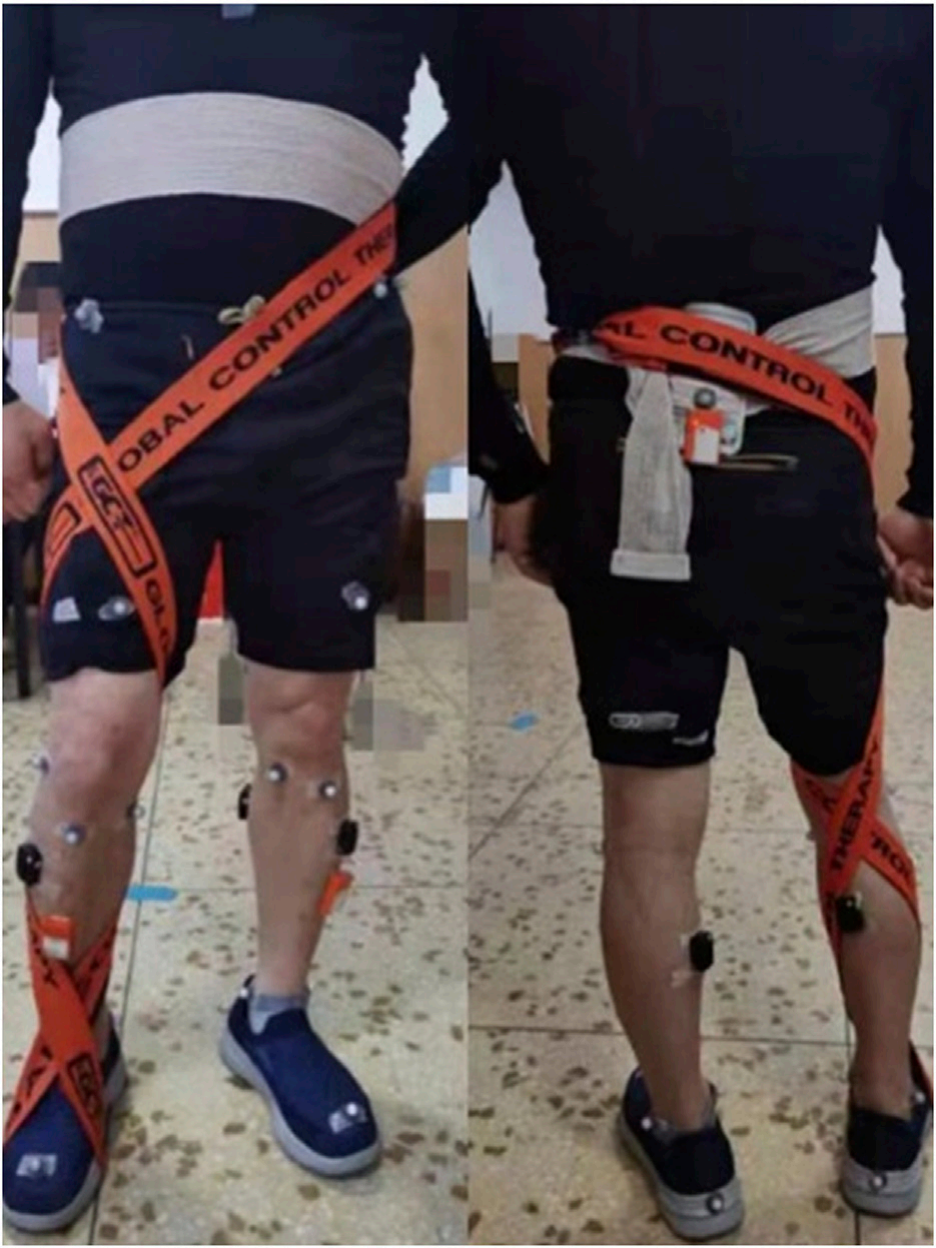
Schematic diagram of lower limb anterior rafting strap binding technique.

The participants were strapped by a hospital rehabilitation therapist, and the specific method refers to the approach of Wang et al., (2018) with some adjustments. The method is as follows: The patient sat and bent his or her hip and knee, took an elastic strap with a width of 5 cm, placed the middle of the strap on the bottom of the affected foot, and crossed it in front of the ankle joint. Then, the patient was asked to stand up, the strap was wrapped around the back of the shank, then around the back of the knee joint, and wound up along the thigh of the affected side. It was crossed in front of the thigh and continued to be wrapped around the back of the waist to be tied and fixed. The tension of the strap was adjusted to a comfortable level for the patient during the tying process. The specific strapping method is shown in Figure 1.

#### 2.2.2 Data collection and reduction

The 6-lens Oqus 400 infrared motion capture system (Qualisys, Sweden, 200 Hz) was used to collect the trajectory data of body surface landmarks during a walking distance of 5–7 m from the starting point of the participants.

The raw 3-D trajectories of reflective markers were smoothed using a Butterworth low-pass filter with a cutoff frequency of 13.3 Hz to reduce random noise (Yu et al., 1999). A gait cycle was defined as the time from when the affected foot landed on the ground until it landed again, and the support phase was defined as the time from when one foot landed on the ground until that same foot was lifted off the ground. Step length and step width were calculated using the toe coordinates. Step length was defined as the distance between the unaffected side foot hitting the ground and the affected side foot hitting the ground in the anteroposterior direction, while step width was defined as the distance between the left and right directions of two consecutive heel strikes. The Euler angles method was employed to calculate the three-dimensional angle of each lower limb joint (Wu et al., 2002). The proportions of the stance phase of the unaffected and the affected sides were calculated, representing the time of the stance phase of one limb in relation to the whole gait cycle. The ratio of the unaffected-to-affected side stance phase was also calculated. The cubic spline interpolation method was used to standardize the angle of each lower extremity joint into 101 data points in one gait cycle, facilitating subsequent statistical analysis. Step length and width were standardized by dividing leg length (LL), defined as the distance from the greater trochanter of the femur to the ipsilateral lateral malleolar. All results were averaged by three valid datasets.

#### 2.2.3 Data analysis

The dependent variables (step length, step width, the proportion of stance phase on the unaffected side, the proportion of stance phase on the affected side, unaffected–to–affected side stance phase ratio) were compared before and after binding using a paired t-test in SPSS 21.0 software (SPSS, Chicago, IL, United States). Ensemble averages of the angles of hip flexion and extension, hip abduction and adduction, hip internal rotation and external rotation, knee flexion and extension, ankle dorsiflexion and plantar flexion, ankle inversion and eversion, ankle internal rotation and external rotation during the gait cycle before and after binding were analyzed using one-dimensional SPM paired sample t-test (Pataky et al., 2013). All SPM analyses were implemented in MATLAB R2013a (The MathWorks Inc., United States) using open-sourced code.

The significance level was set at 0.05. The effect size was represented by the Cohen’s d. The values d = 0.2, d = 0.5, and d = 0.8 correspond to small, medium, and large effect sizes, respectively (Cohen, 2013).

## 3 Results

### 3.1 Spatio-temporal parameters

The results of the statistical analysis (Table 2) indicate that the standardized step length decreased, and the standardized step width increased after strap binding compared to the unbound condition. There were no statistically significant differences in the proportion of stance phase on the unaffected side, the proportion of stance phase on the affected side, and the unaffected-to-affected side stance phase ratio before and after the strap binding.

**TABLE 2:** Spatiotemporal parameters of participants’ gait before and after lower extremity elastic strap binding technique.

parameters	unbundled	after binding	t value	*P*-value	*Cohen’s d*
step length (LL)^*^	0.49 ± 0.19	0.47 ± 0.18	2.396	0.024	0.108
step width (LL)^*^	0.33 ± 0.08	0.34 ± 0.08	2.838	0.008	0.125
the proportion of stance phase on the unaffected side (%)	64.48 ± 7.41	64.14 ± 7.08	0.422	0.676	0.047
the proportion of stance phase on the affected side (%)	72.38 ± 9.38	73.38 ± 8.94	1.128	0.269	0.109
unaffected-to-affected side stance phase ratio	1.13 ± 0.16	1.14 ± 0.13	2.048	0.292	0.069

Note: LL, leg length, normalized relative to leg length, * indicates comparison before and after strap binding, *P* < 0.05.

### 3.2 Joint angles

**FIGURE 2:**
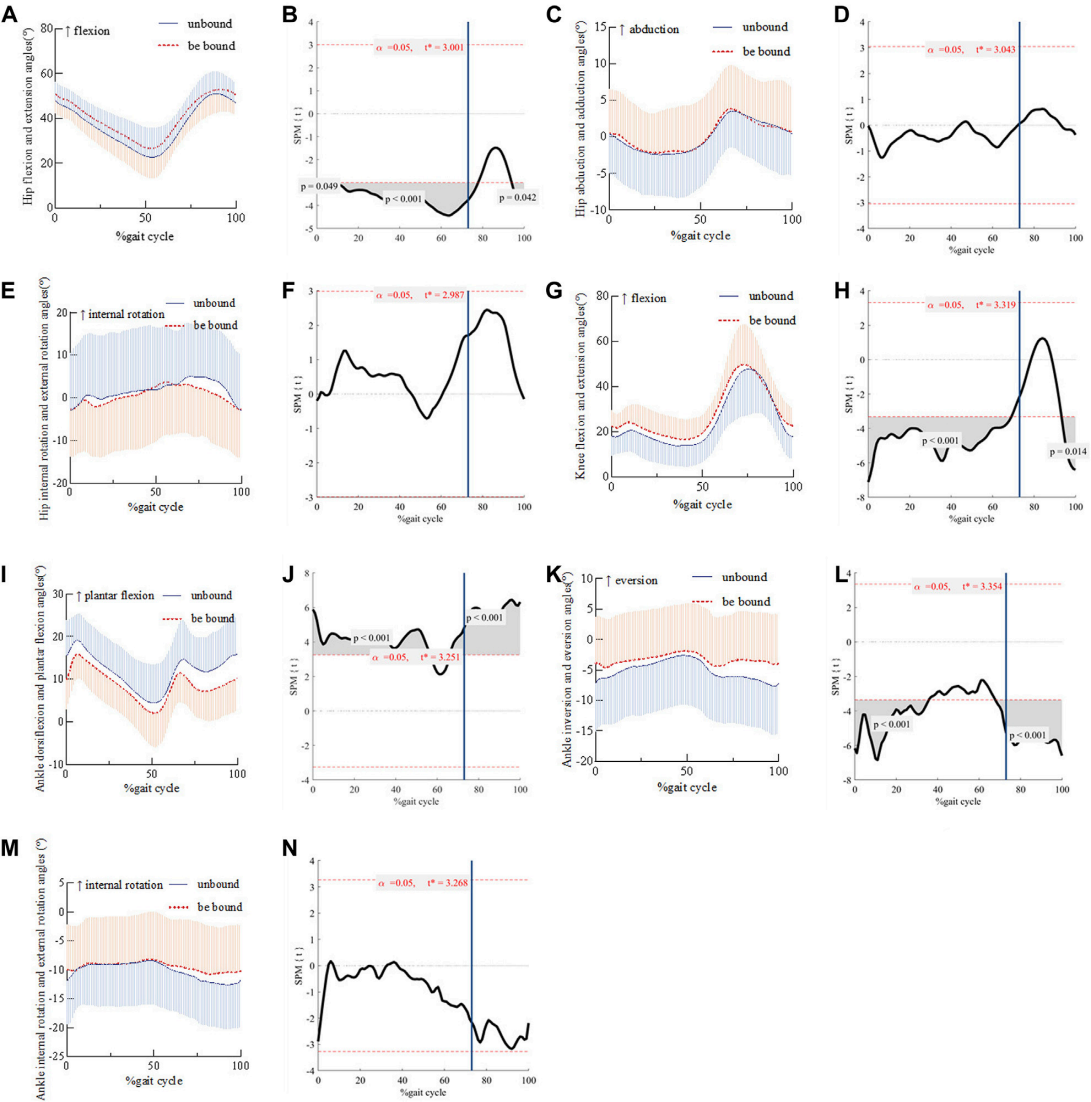
Comparison of hip flexion and extension angles and their SPM during gait cycles before and after strap binding Panel 2 **(A, C, E, G, I, K, M)** shows the change of joint angles during the gait cycle. **(B, D, F, H, J, L, N)** is the SPM(t) value, and the red dashed line indicates the critical threshold. The blue vertical line represents the moment when the affected limb leaves the ground. When the SPM(t) value exceeds the critical threshold, the difference before and after binding is statistically significant (*P* < 0.05).

The flexion angle of the affected hip joint in the three stages of the gait cycle (0%–2%, 7%–77%, and 95%–100%) after strapping was larger than that before binding (Figure 2A, B). The hip abduction and adduction angles (Figure 2C, D) and the hip internal rotation and external rotation angles (Figure 2E, F) showed no significant changes before and after strapping. The knee flexion angle of the affected side in the two stages (0%–69% and 94%–100%) during the gait cycle of the patients after strapping was larger than that before strapping (Figure 2G, H). The ankle plantar flexion angle of the affected side in the 0%–57% and 67%–100% stages of the gait cycle after the strapping was smaller than that before binding (Figure 2I, J). The ankle inversion angle of the affected side in the 0%–35% and 68%–100% stages of the patients’ gait cycle after strapping was smaller than that before binding (Figure 2K, L). The ankle internal rotation and external rotation angles (Figure 2M, N) showed no significant changes before and after strapping.

## 4 Discussion

The evaluation of gait kinematics is crucial for the diagnosis, treatment and rehabilitation of stroke (Zhang et al., 2023). In this study, infrared motion capture technology combined with SPM was used to analyze the immediate kinematic changes of lower extremity elastic strap binding technique on the gait cycle of stroke patients. The results showed that this technique decreased the standardized step length and increased the standardized step width during walking, increased hip flexion angles, knee flexion angles and decreased ankle plantar flexion angles. Thus, the experimental results partially supported the research hypothesis.

In this study, it was found that the lower extremity elastic strap binding technique could decrease the step length and increase the step width of stroke patients. Research has shown that the human body can improve dynamic stability by shortening the step length, bringing the center of mass closer to the support plane (Yang et al., 2016), and by increasing the step width to expand the support base. The lower extremity elastic strap binding technique stabilizes the joints of the lower limbs through the elastic characteristic of the strap band, limits excessive extension of the lower limb joints, decreases the step length, and increases the step width of the patients, resulting in a more stable gait (Hak et al., 2013; Yang et al., 2016). This is crucial information for rehabilitation practitioners and caregivers, as it allows them to tailor rehabilitation plans based on these changes, aiding patients in regaining their walking abilities and reducing the occurrence of accidents. However, the Cohen’s d value is small, as the degree of change in step length and width is only affected to a small extent by the elastic characteristic of the strap band.

The lower extremity elastic strap binding technique can increase the hip flexion angles during walking in stroke patients. Previous studies have indicated that stroke patients have limited hip movement due to weak hip flexion strength on the affected side (De Quervain et al., 1996), significantly affecting walking ability and walking speed (Kim and Eng, 2004). In this study, the strap was crossed at the front of the affected thigh and wrapped around the back of the patient’s waist for fixation. The elastic characteristic of the strap was used to assist hip flexion, which increased the hip flexion angle during stance phase of the patient’s gait cycle.

The lower extremity elastic strap binding technique increases the knee flexion angles in stroke patients during walking. This study found that knee flexion angle increased during stance phase of the patient’s gait cycle, confirming the effectiveness of wearing a strap to decrease knee hyperextension and the stiff gait pattern of the knee joint caused by muscle rigidity in stroke patients during walking. This may be attributed to the fact that the back of the affected knee joint is wrapped with the strap, providing assistance for knee bending on the affected side. Moreover, as there is a positive correlation between ankle dorsiflexion and knee flexion (Kim and Won, 2019), the resistance to plantar flexion exerted by the strap on the ankle may further increase the knee flexion angle during landing (Choo and Chang, 2021).

Anterior lower limb rafting decreases the ankle plantar flexion angles in stroke patients during walking. In stroke patients, due to weakness of the ankle dorsiflexors and evertors, or excessive activity of the plantar flexors and invertors, the foot cannot be fully lifted during the walking swing stage, sometimes accompanied by excessive inversion, which is manifested as foot drop (Prenton et al., 2016), one of the main reasons affecting gait (Choo and Chang, 2021). The elastic strap binding technique used in this study stabilizes and supports each joint through the elastic characteristic of the strap, fixes the position of the limb, and supports and stabilizes the weak muscle groups (Liu et al., 2022). It can be regarded as the combination technology of elastic band orthotics that acts on the ankle, knee, and hip. Compared with ordinary ankle-foot orthotics that restrict the patient’s active dorsiflexion ability of the ankle joint and their control of movement, elastic band orthotics have less restriction on the active movement ability of the ankle joint (Xie et al., 2013). By providing appropriate plantar flexion resistance to the feet (Kim and Won, 2019), it effectively prevents the foot drop thus reduces the risk of falling in stroke patients. The strap was interlaced in front of the affected limb’s ankle, and appropriate resistance to plantar flexion and inversion was applied to the patient’s foot. The results showed that the plantar flexion angle and inversion angle of the ankle of the patients were reduced during most phases of the gait cycle, confirming that the technique could assist the patients in dorsiflexion and eversion of the ankle during walking, thus improving the abnormal gait.

The lower extremity elastic strap binding technique can improve the abnormal gait of stroke patients and play an auxiliary role in gait rehabilitation. Due to dysfunction, stroke patients often inhibit the use of the affected limb, resulting in insufficient use of the affected limb and loss of behavioral and neuronal function (Maier et al., 2019). Rehabilitation of stroke patients may be closely related to neural remodeling, which requires enhancing existing synaptic conduction and creating new connections (Szelenberger et al., 2020). Neuroscience studies have shown that the functional results of neuroplasticity are task-specific and depend on the nature of training (Yeung et al., 2018). This means that, in order to improve the ability to walk independently, stroke patients should relearn movement patterns by practicing walking repeatedly (Mayr et al., 2007). By making stroke patients adapt to the coupling of the walking process and being guided by the goal of normal walking, the strap binding technology produces higher activity in the sensorimotor area. It assists stroke patients in obtaining a more normal gait pattern and provides proprioceptive feedback to stimulate changes in the excitability of the motor cortex (Yeung et al., 2018). This is more conducive to the gait rehabilitation of stroke patients. Given the potential of lower extremity elastic strap binding technique to ameliorate issues of foot drop and restricted hip and knee flexion in stroke patients, in forthcoming clinical applications, rehabilitation therapists may consider employing lower extremity elastic strap binding technique for gait rehabilitation training specifically tailored for stroke patients exhibiting symptoms of foot drop and limited hip and knee flexion.

## 5 Limitations

In the testing of this study, a non-randomized approach was used, where all participants first underwent walking tests without binding, followed by walking tests with binding. This may introduce systematic bias. Additionally, we predicted an effect size of 0.4 for the impact of binding, which was used to calculate the sample size. However, the actual effect size was lower than 0.4, potentially leading to an underestimation of the sample size chosen, constituting one of the limitations of this study.

## 6 Conclusion

The lower extremity elastic strap binding technique can decrease the hip flexion and knee flexion limitations in stroke patients during walking, and reduce the ankle plantar flexion and ankle inversion angle of stroke patients. The lower extremity elastic strap binding technique can help stroke patients adopt a more stable gait pattern.

## Data Availability

The datasets presented in this study can be found in online repositories. The names of the repository/repositories and accession number(s) can be found below: 10.6084/m9.figshare.25922287.
